# Classification of Parkinson’s disease and isolated REM sleep behaviour disorder: delineating progression markers from the sebum volatilome

**DOI:** 10.1038/s41531-025-01026-8

**Published:** 2025-07-15

**Authors:** Caitlin Walton-Doyle, Beatrice Heim, Eleanor Sinclair, Sze Hway Lim, Katherine A. Hollywood, Joy Milne, Evi Holzknecht, Ambra Stefani, Birgit Högl, Klaus Seppi, Monty Silverdale, Werner Poewe, Perdita Barran, Drupad K. Trivedi

**Affiliations:** 1 Michael Barber Centre for Collaborative Mass Spectrometry, Manchester Institute of Biotechnology, Department of Chemistry, University of Manchester, Manchester, M1 7DN UK; 2 Department of Neurology, Innsbruck Medical University, Innsbruck, Austria; 3 Department of Neurology, Salford Royal Foundation Trust, Manchester Academic Health Science Centre, University of Manchester, M6 8HD Manchester, UK

**Keywords:** Parkinson's disease, Predictive markers, Diagnostic markers, Metabolomics

## Abstract

Parkinson’s Disease (PD) has been associated with a distinct odour, emanating from the skin and strongest in sebum-rich areas. Here, volatile components from sebum were analysed directly with Thermal Desorption Gas Chromatography - Mass Spectrometry. We analysed samples from subjects with clinically established PD (*n* = 46), controls (*n* = 28) and participants with isolated REM sleep behaviour disorder (iRBD, *n* = *9*) to investigate metabolite changes in probable prodromal PD. We found 55 significant features where abundance from individuals with iRBD was intermediate between PD and control and assigned putative identifications. Olfactory analysis of the iRBD samples showed three classified as PD, of which two displayed PD symptoms on clinical follow-up. Further, we analysed PD participants sampled at yearly intervals and investigated features displaying regulation over the visits. Our findings support the use of sebum as an accessible biofluid, rich with measurable volatile compounds that alter in abundance in individuals with PD and iRBD.

## Introduction

Parkinson’s Disease (PD) is the second most common neurodegenerative disorder, with more than six million people affected worldwide and an expected two-fold increase in prevalence over the next generation^1^. The pathological hallmarks are neuronal α-synuclein positive inclusions and cell loss in the substantia nigra, other areas of the brain, and the peripheral autonomic nervous system causing the cardinal motor features of bradykinesia, rigidity and rest tremor as well as a plethora of non-motor symptoms^2^. Current diagnostic criteria for PD are anchored on clinical features, which overlap with other conditions and may result in initial misclassification^3^. Highly sensitive and specific tests, therefore, are a major need in PD - both to enhance diagnostic accuracy and to enable detection of the earliest disease stages where future disease-modifying therapies would be the most effective^3^.

Clinically manifested PD evolves from a prodromal stage characterised by the occurrence of a variety of non-motor symptoms, including anosmia, constipation, anxiety, depression, change in cognitive behaviour as well as REM-Sleep Behaviour Disorder (RBD)^4,5^. Together with various risk markers, these have been used to define research criteria for prodromal PD. Within these criteria a polysomnographic diagnosis of isolated RBD (iRBD), characterised by loss of REM-atonia and jerks or dream-enacting behaviours during REM sleep confers the highest risk for future PD with a likelihood ratio of 130^6^. In order to diagnose RBD, polysomnography (PSG) is needed to demonstrate the presence of REM sleep without atonia, which is an excessive electromyographic activity during REM sleep^6^.

PD has been associated with a distinct odour, strongest in sebum-rich areas. Sebum is a lipid-rich substance secreted by the sebaceous glands on the skin, which are most concentrated on the face and upper back. Increased sebum output (seborrhoea) and seborrhoeic dermatitis are well-known features of PD and changes in volatile profiles of sebum have recently been detected using Thermal Desorption Gas Chromatography – Mass Spectrometry (TD GC-MS)^7,8^. Sebum is an alternative biofluid found on the skin and that can be obtained by non-invasive sampling, giving rise to advantages such as at-home sampling as well as low consumables and transport costs. It has been shown that sebum does not require the same cryogenic storage as other biofluids to maintain its diagnostic capabilities, further reducing costs associated^9^.

To date, TD GC-MS has not been employed on cohorts showing prodromal PD symptoms, despite evidence that the change in odour occurs premotor symptoms. This motivates our investigation to determine if volatile components of sebum have potential for biomarkers for early diagnosis. In this study, we analysed sebum samples from PD and control cohorts, finding good discrimination between their volatile metabolite profiles, adding to our body of work in this area and improving our discrimination between disease and control. We investigated features that contributed highly to the separation of phenotypes and putatively identified them using fragment spectra. We further analysed samples from iRBD participants. We describe how odour and mass spectrometry markers permit classification between these three different phenotypes, and features where iRBD concentration lies between disease and control, indicative of possible biomarkers of early stages of disease. Further, we examined markers in samples collected longitudinally from PD participants at three annual timepoints to investigate possible progressive features.

## Results

### PD classification using Sebum VOCs (PwPD *vs*. control)

**Fig. 1:**
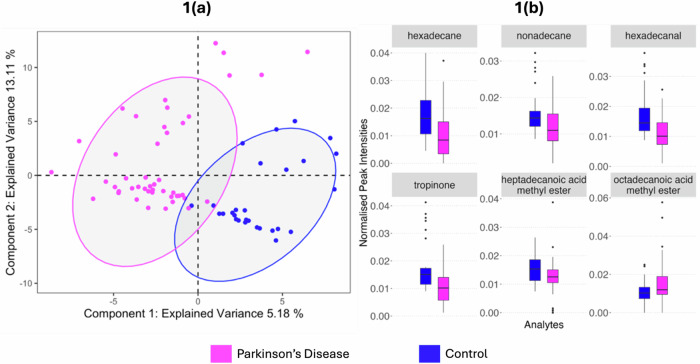
Modelling VOC profiles from skin swabs of Parkinson’s disease and control participants. **a** Optimised sPLS-DA shows two clusters distinctly separating PD (pink) and controls (blue), validated by 3-fold cross validation with 100 repeats. **b** Example box plots for six of the 85 significant features, chosen to demonstrate changes in mean expression in PD and controls, selected using VIP scores (>1) and cross-validation feature stability scores (>0.8). For further details on the selection of these features, see supplementary information.

Following deconvolution, the resultant data matrix contained 613 robust features. The sPLS-DA scores plot (Fig. 1a) shows good clustering of the two phenotypes, which was cross-validated and had an error rate of 0.2. We have removed the odour port from our TD GC-MS instrument for this work, which resulted in a higher signal (by one order of magnitude from our earlier work)^7,8^, and this reveals more features from the samples, some of which are more robustly diagnostic. Of the 613 features, 85 were determined to be significant variables (VIP > 1, stability > 0.8) that enable classification between PD and control. When investigating regulation, 25 of the significant variables are seen to be upregulated with PD and 60 downregulated. Of the significant features, 28 could be assigned putative identifications at metabolomics standards initiative (MSI) level 2: seventeen as alkanes and seven as fatty acid methyl esters (FAMEs). Purine, tropinone, oleamide and hexadecanal were also identified and upregulated in the control samples. Lack of identification of the remaining significant features can be attributed to the lack of a suitable match in spectral libraries and absence of precursor ions, or possibly due to chromatographic co-elution. Example expressions of annotated significant features are displayed in Fig. 1b. These data validates our earlier observation that volatile compounds from sebum can distinguish PD from control, irrespective of the geographical location of subjects. A list of all features found significant is shown in the supplementary information Table S1.

### Classification of iRBD from control and PD

**Fig. 2:**
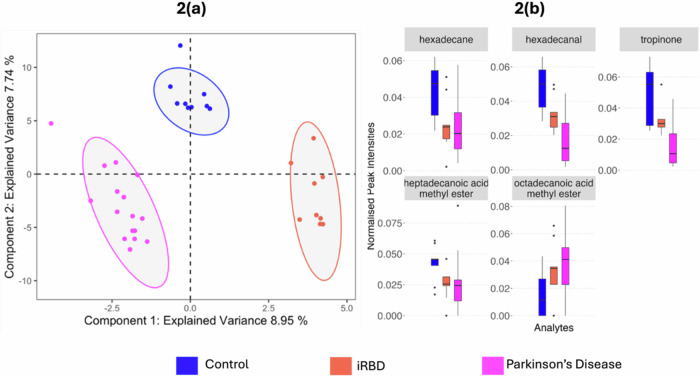
Modelling VOC profiles collected from skin swabs of PD, control and iRBD participants from the Austrian cohort. **a** Optimised sPLS-DA show three clusters distinctly separating PD (pink), controls (blue) and iRBD (orange) participants, validated by 3-fold cross validation with 100 repeats. **b** Examples of significant features selected using VIP scores (>1) and cross-validation feature stability scores (>0.8) to compare mean expression in PD, controls and iRBD are shown with box plots. For further details on the selection of these features, see the supplementary information.

We examined the cohort collected from one site (Austria) using all 613 features from the data matrix. The scores plot of the sPLS-DA (Fig. 2a) shows 3 distinct groups (cross-validated with an error rate of 0.06). The control participants cluster the most tightly and thus show the least variability between individuals, while more within-group variability is seen in iRBD and PD. Examples of putatively identified features displaying mean iRBD regulation between that of PD and control groups are displayed in Fig. 2b.

Of the 613 features, 76 features had VIP score >1 (within components 1 and 2) and cross-validation feature stability score > 0.8 and thus were considered significant. Out of these 76 features, 55 showed the mean iRBD concentration to be between that of PD and control, suggesting possible progression features. Of these 55 possible progression features, 19 could be putatively identified (MSI level 2) and these were: 7 alkanes (different regulations), 8 FAMEs (different regulations), two annotations of tropinone (upregulated in control), an aldehyde (upregulated in control) and purine (upregulated in PD). Examples of those annotated features are shown in Fig. 2b.

### Odour analysis of iRBD

All iRBD samples, along with three PD and three control samples were subjected to odour analysis as described previously^10^. The hyperosmic individual (HI), who was not told how many of each phenotype were presented, was able to distinguish PD from control in all cases and also classify six of the iRBD samples separately as possessing a distinct odour. Three of the iRBD samples were assigned as PD by the HI. Two out of the three iRBD participants classified as PD showed signs of conversion to PD at clinical follow-ups post-sampling. This observation indicates that the odour of each disease can be distinguished and that iRBD has a distinct volatilome containing volatile markers that may report on the conversion/progression to PD.

### VOC signature with Parkinson’s disease progression – longitudinal PD samples

**Fig. 3:**
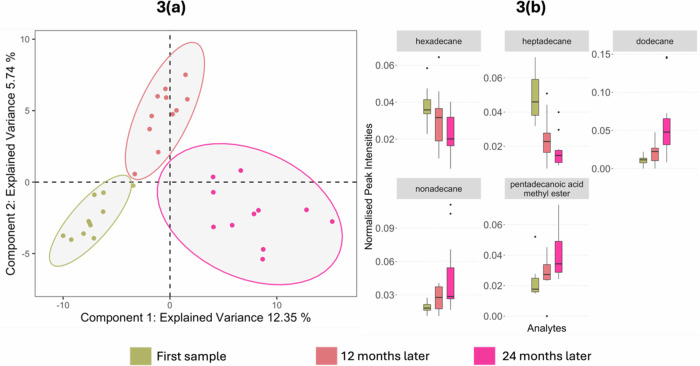
Modelling VOC profiles collected from skin swabs of PD participants from the UK cohort over three consecutive years. **a** Optimised sPLS-DA displays three clusters distinctly separating the time points in a trajectory from left to right, validated by 3-fold cross validation with 100 repeats. **b** Example significant features that exhibited increasing or decreasing trend as time progresses; selection made using VIP scores (>1) and cross validation feature stability scores (>0.8) to compare mean expression in PD over time. For further details on the selection of these features see supplementary information.

When comparing the volatile features found from the same individuals sampled at yearly intervals, the skin volatilome exhibits good separation determined by cross validated (error rate of 0.15) sPLS-DA (Fig. 3a). The intragroup variation increases over time, with tighter clustering seen at the first year of sampling, indicating higher variation between individuals as PD progresses. Due to the separation of the timepoints on both components 1 and 2, features with VIP > 1 in either component, as well as stability score >0.8 were investigated and considered significant. A total of 38 features were selected for further analysis and annotation.

Of the 38 significant features, 22 displayed regulation in abundance over the visits, meaning after 12 months, the abundance was between that found at the time of initial sampling and at 24 months from sampling (Fig. 3b). Interestingly none of the features found as significant in classifying PD (Fig. 1 and above) were significant and regulated over the timepoints, implying that the progression features are distinct from diagnostic features. Five of the features that displayed regulation could be assigned a putative identification: four as alkanes (different regulations) and one as a FAME (increasing over the three visits) (Fig. 3b).

We sought to determine if these progression features were due to any confounding effects such as age, gender, BMI, medication or comorbidities. We adjusted for known confounders, including them as variables within our models. Further, we used variables to predict these confounders to ascertain the specificity of these measured metabolites to disease phenotype (Tables S1–S4). All confounders ranked below all metabolites, while gender, high cholesterol and heart conditions ranked in the bottom half of the ranking list (Table S2a and S2b). Additionally, none of the models using significant features could predict confounders as an outcome (Table S3,) which indicates that these significant features are specific to the phenotypes classified, not any potential confounders. We found no strong correlations (>0.5) between significant features and drug intake and the measured volatilome was not predictive of any clinical characteristic or drug intake (Table S2c and S4). These analyses overall suggest that the volatilome measured in this study is representative of the molecular phenotype of Parkinson’s disease and of iRBD and is independent of other clinical history and medication.

## Discussion

We investigated and interpreted the features of interest in all three models using putative annotations from spectral matches with compound libraries at MSI level 2 confidence^11^. Analysing lipid standards by GC-MS, including headspace TD GC-MS gives rise to several resolvable features, many of which are putatively identified as alkanes^8^. This indicates that substantial fragmentation occurs prior to GC separation^8^. This degradation of lipid molecules may occur in either the headspace analysis (incubation and extraction) or upon introduction to the GC. The breakdown of lipids combined with the variation in branching and double bond position in hydrocarbon chains of lipid tail groups can result in many isomers identified as different features^8,12^. Further, the harsh ionisation and fragmentation processes of GC-MS can result in a lack of a molecular parent ion, thus, molecules with hydrocarbon chains are often identified by their fragmentation fingerprint and carbon chain length can be difficult to annotate.

We therefore hypothesise that the alkanes and FAMEs found with high VIP and stability scores in models here are derived from larger lipids in sebum. Changes in fatty acid concentrations in brain tissue^13^, blood^14^ and the gut^15^ have been associated with PD, and we have previously reported alterations in sebaceous lipids as features of interest in PD^16,17^. Fatty acids are a well-documented product of the breakdown of larger lipid molecules such as acylglycerols^18^^,19^. Similarly, aldehydes in headspace may arise due to oxidation of lipids, either endogenously or during the analysis^20^. The inclusion of these as significant in the longitudinal study indicates that lipids also alter with disease progression.

Tropinone was identified as significant in the PD *vs*. control model, and significant with regulation in the iRBD model, the latter of which had two features assigned. It was observed to decrease in PD and show iRBD as the intermediate concentration. It is an alkaloid derived from putrescine^21^. Studies in animal models have shown an important role of putrescine in skin physiology and neuroprotection^22^. Oleamide, an endogenous fatty acid, was also observed to be downregulated in PD compared to control. It is a sleep-inducing lipid known to decrease motility in animal models^23,24^, although interestingly, it was not found to be a feature of interest when classifying iRBD.

Two features annotated as purine were found to be significant. One was upregulated in control, and a second feature was found with intermediate expression in iRBD and downregulated in control (Fig. S1). The differences in regulation are likely due to two features, with purine-like fragmentation being annotated with the same identification. We hypothesise these are purine derivatives and differences in regulation are indicative of pathway dysregulation, manifesting as an increase in some features of purine metabolism and a decrease in others. One of the end products of purine metabolism is urate, an antioxidant that has been described as a risk marker for PD, and lower levels are also associated with higher severity of disease^25–27^. Alterations in the purine pathway have been researched in investigations for PD biomarkers, as well as in comparison with other neurological dieases^28–30^.

The overall volatilome of iRBD having many components seen as intermediary between PD and control, lends further support to sebum as a reservoir for diagnostic biomarkers of PD. This is a discovery study showing great potential and will be validated with a larger sample set. As iRBD can evolve into different disorders (PD, Dementia with Lewy Bodies, Multiple System Atrophy), future work will focus on which of the iRBD-associated features observed in this study are most specific for conversion to these conditions.

To conclude, this study demonstrates the ability of TD GC-MS to discriminate the volatilome of PD from control using sebum collected on gauze swabs, followed by supervised multivariate analysis. When investigating significant features that contribute to classification, we find many metabolite features that can be attributed to lipid breakdown products, validating our previous findings^8^.

Using the same technique and analysing sebum from individuals with iRBD, we observe that a differential volatilome in these participants is present, providing potential for measurement of prodromal molecular markers. Significant features detected showed the iRBD cohort with a mean concentration between PD and control samples, indicative of disease progression. Finally, we analysed the volatile metabolites in the headspace of samples taken at yearly intervals to investigate changes over disease course and can demonstrate a clear classification of samples based on the stage of disease. The significant features again are putatively annotated as hydrocarbons and FAMEs, supporting the hypothesis of differential regulation of lipids.

Such putatively identified markers limit the opportunity for detailed pathway analyses, but certainly furthers our hypothesis that the abundance of lipids and other metabolites captured in skin swabs from areas rich in sebum change significantly in the course of Parkinson’s disease^8^. We have shown that dysregulation of lipids can be observed in swabs from iRBD and in longitudinally sampled participants with PD (PwPD). We have previously demonstrated that liquid chromatography-mass spectrometry (LC-MS) can be used to further investigate these larger molecules^17^ and here show the power of direct analysis of the volatilome. With complementary analytical platforms we can better understand the mechanisms and pathways causing these changes as well as identify features that differentiate between disease and control which would be suitable for targeted MS analysis akin to that used routinely for newborn screening where more than 30 M tests are conducted globally p/a^31^ as well as common and increasing use of mass spectrometry in drug discovery, clinical trials, microbiology, endocrinology and therapeutic drug monitoring^32^.

We have established a method using facile and non-invasive sebum sampling to demonstrate differential volatile signatures for PD, iRBD, and control participants. We also describe changes in the volatilome as the disease progresses and offer a route to discover markers that could be used to inform the development and efficacy of new treatments. Together, these findings offer a novel approach for early PD classification, where current robust clinical tests are lacking, as well as offering discrimination over disease progression.

## Methods

### Sample collection and clinical cohort

Subjects were recruited at two sites: the Movement Disorder and Sleep Disorder out-patient clinic, at the Department of Neurology, Medical University Innsbruck, Austria (Austria cohort) and the Department of Neurology at Salford Royal Foundation Trust (UK cohort). Clinic staff, patients’ caregivers, and patients without neurodegenerative or ongoing dermatological disorders were invited to participate as healthy controls. Sebum samples were collected from the upper back of participants using sterile cotton-based gauze swabs (7.5 × 7.5 cm) by single individuals to avoid variance due to sampling. The sampling protocol is described in earlier work, although in this study, samples were taken at two sites from well-studied cohorts with refined instructions and identical for sampling^7,8^.

The UK cohort consisted of people with PD (PwPD) (*n* = 30) and control (*n* = 19) participants. Of 30 PD participants, 11 were sampled longitudinally three times in total, ~12 and 24 months after the first sampling. Controls were not included in longitudinal sampling. The Austrian cohort consisted of participants diagnosed as PwPD (*n* = 16) and PSG-confirmed iRBD (*n* = 9)^6^ as well as controls (*n* = 9). Participant demographics are detailed in Table 1, all PD participants were diagnosed by a Movement Disorder specialist, using the UK Brain Bank Criteria^33^.

**Table 1:** Participant demographic information and sample groups for analyses

Location/phenotype	Participants	Sex (M:F)	Age mean (SD)	BMI mean (SD)
UK/ PD Timepoint 1	30	5:1	63.83 (7.87)	27.01 (3.67)
UK/ PD Timepoint 2 & 3*	11	11:0	60.09 (8.51)	27.42 (2.78)
UK/ Controls	19	10:9	63.89 (11.19)	26.37 (3.87)
Austria/ PD	16	3:1	64.63 (6.38)	25.77 (3.78)
Austria/ iRBD	9	8:1	69.89 (11.83)	27.93 (4.69)
Austria/ Controls	9	8:1	60.44 (4.80)	27.06 (3.61)

^*^consisted of same 11 PD participants sampled at ~12 and 24 months after first sampling.

For age and BMI, the standard deviation (SD) is shown in parentheses.

Two iRBD subjects developed subtle Parkinsonian signs after follow-up but had been free of any such sign at the time of sampling. The majority of our PD and iRBD participants are male, in line with the global prevalence rates^34,35^.

All participants provided informed consent for this study. Ethical approval for this project (IRAS project ID 191917) was from the NHS Research Authority (REC reference: 15/SW/0354) and from the Medical University of Innsbruck (reference: AN1979 336/4.19 401/5.10 (4464a)).

Following collection, the gauze swabs were placed in Ziplok plastic bags, transported to the test site and stored at −80 °C until analysis. It has previously been shown that storing samples at ambient temperatures does not affect the capability of classifying PD, and thus the time spent out of cryogenic storage in collection and postage is not considered damaging to the validity of the samples here^9^. Dynamic headspace was analysed from all these samples in a randomised order. Raw data were deconvolved, annotated and converted to a data matrix consisting of 613 features. We interrogated these data. In summary, we sought to answer two questions:

1. Do components that contribute to the skin swab VOC signature alter as Parkinson’s progresses?
2. Is there an VOC signature associated with iRDB and can it be distinguished from that of PD and of control participants, using TD GC-MS?

### Materials

Sterile gauze swabs (Arco, UK) and sample bags (GE Healthcare Whatman, UK) were used for all sample collection. The headspace was collected into 20 mL headspace vials (GERSTAL, Germany) and absorbed onto TENAX TA sorbent tubes and packed liners (GERSTAL, Germany). The solvents used for making system suitability mix were Optima LC-MS grade methanol (Fisher Scientific) and CHROMANORM absolute 99.8% ethanol (VWR Chemicals). The system suitability mix used to check for instrumental drift was a mix of seven compounds (Sigma-Aldrich): l (-)-carvone (27.0 μM), δ-decalactone (96.4 μM), ethyl butyrate (150.5 μM), ethyl hexanoate (30.5 μM), hexadecane (43.7 μM), nonane (83.07 μM), and vanillin (100.8 μM), all in MeOH: EtOH (9:1).

### Analytical setup

Samples were thawed and removed from bags before transference to headspace vials (20 mL). Analysis was performed on an 7890 A GC (Agilent) coupled with a 5975 MSD (Agilent) equipped with a GERSTAL MPS handling system. Volatile analytes were preconcentrated by incubating samples at 80 °C for 10 mins. Analytes were trapped using 1 L dry nitrogen purged through the headspace at 70 mL/min and onto the TENAX TA sorbent tube held above at 40 °C. The sorbent tube subsequently was transferred to the Thermal Desorption Unit (TDU) located in the GC inlet where it was held at 30 °C for 1 min before the temperature increased at 12 °C/s until 280 °C and held for 5 min. The Cooled Injection System (CIS) was held at 10 °C for 0.5 min before a 12 °C/s ramp up to 280 °C and held for a further 5 min. Analytes were separated on an Agilent VF-5MS column (30 m x 0.25 mm × 0.25 um) with a flow of 1 mL/min and a split ratio of 1:20. The temperature of the oven was held at 40 °C for 1 min then ramped at 25 °C/min to 180 °C, 8 °C/min to 240 °C and held for 1 min then 20 °C/min to 300 °C where it was held for 2.9 min. Positive pressure was added to the splitter plate at 1.6 mL/min. The MSD operated in scan mode between *m/z* 30-800 with the source temperature held at 230 °C and quadrupole temperature held at 150 °C.

### Olfactory analysis

Samples (gauze swabs in plastic bags) of known phenotype were presented to the tester (an individual with hyperosmia - a heightened sense of smell) to train and familiarise. Once training was completed, blinded samples were presented to the tester in the presence of observers. The tester was asked to describe the strength and type of smell, which was recorded on a ranking scale with comments. The tester ‘reset’ their nose by smelling a scent refresher of their choice between samples and periodic breaks were taken if the tester was overwhelmed. A subset of samples was revisited at the tester’s request.

### Data processing and statistical analyses

Raw mass spectrometry data files were converted to mzXML open format using ProteoWizard MSconvert^36^. Subsequently, mzXML files were deconvolved in R using the eRah package^37^ resulting in a data matrix with 613 deconvolved and putatively identified features. The features were assigned putative identifications using the GOLM and NIST 14.0 databases within eRah. Assignments of TMS derivatives, siloxanes or features with a match factor < 70 were not considered and removed from further analyses. The final data output was a matrix of features and the relative intensity of these in each sample. In the absence of biological pooled QC, a mixture of standards was injected five times at the start of each batch, after every five sample injections and three times at the end of each batch. The instrument’s stability was assessed using the relative standard deviation (%RSD) of the peaks from this system suitability mix. All peaks of the SST showed %RSD < 20% and data quality was considered satisfactory. All data were normalised to the sum of the total ion count, log transformed and auto-scaled prior to statistical analyses. We employed multivariate statistical analyses methods to analyse the mass spectrometry data. Sparse partial least squares discriminant analysis (sPLS-DA) was used to model data classifying between the studied cohorts described in the Table 2.

**Table 2:** Description of three models generated to compare phenotypes and samples included in each

Comparison	PD	iRBD	Control
PwPD vs. Control (Fig. 1)	Age 64.11 (7.32) BMI 26.58 (3.72) Gender (37:9)	N/A	Age 62.79 (9.64) BMI 26.58 (3.72) Gender (18:10)
PwPD vs. Control vs. iRBD (Fig. 2)	Age 64.63 (6.38) BMI 25.77 (3.78) Gender (3:1)	Age 69.89 (11.83) BMI 27.93 (4.69) Gender (8:1)	Age 60.44 (4.8) BMI 27.06 (3.61) Gender (8:1)
PwPD progression over 24 months since first sample (Fig. 3)	Age 60.09 (8.51) BMI 27.42 (2.78) Gender (11:0)	N/A	N/A

For longitudinal analysis the age stated is based on that at the first visit. For age and BMI standard deviation (SD) is shown in parentheses. For gender, male-to-female ratio is shown in parentheses.

### Statistical analyses method description

In pilot metabolomics studies, the sample size is often smaller than the number of features detected by high-resolution methods used and the data suffers from a high degree of multicollinearity. Instead of feature selection prior to classification task, sPLS-DA allows us to perform classification and variable selection in a single step. Clustering was determined based on the scores plot and the performance of the model was then determined by comparing balanced error rates of 3-fold cross validation repeated 100 times. The number of components was selected from the initial model and then the number of features per component was determined using a grid search. The final sPLS-DA model used these optimised, reduced components and features per component and the performance was then determined by balanced error rates from cross-validation. Variables contributing to the classification of classes, were selected using variable importance in projections (VIP) scores and stability of the variable during cross-validation. A variable was determined to be important if VIP score was > 1 and if stability was >0.8. These features were called *significant features* in this work. A subset that could be annotated from this feature selection was visualised using boxplots for each model.

### Confounders

We investigated the confounding effects for participants’ age, BMI, gender, alcohol consumption (units per week), smoking habits (cigarettes per week) and existing comorbidities. The common comorbidities within our cohorts were high cholesterol, hypertension, history of skin conditions, bone disease, thyroid conditions and heart conditions. We investigated the likelihood of any confounding effects for all these. To investigate if our significant features were highly ranked due to confounding effects, we performed random forest analyses with and without the confounder as an independent variable. At each node, attributes equal to the square root of the total number of attributes in each data were randomly drawn. We created a separate model adjusting for one confounder at a time and compared it with original unadjusted model. We repeatedly (100 times) split the data in each model where 66% observations were used for training and 34% were used for testing. The final output is based on the majority vote from individually developed trees over 100 repeats on test sets. When a model’s classification accuracy improved by 10% or more in the adjusted model, a confounding effect was concluded. To account for the chances of a smaller sample size or class imbalance within these modes, we limited the depth of individual trees to three, no subset splits smaller than 5 were allowed for any model and we attributed class weights inversely proportional to their frequencies. We also used each confounder as a dependent variable to determine if these significant variables can classify or regress against the confounder, predicting it as an outcome. We used Matthew correlation coefficients (MCC) and regression coefficients (R^2^) to determine if the significant features in all the above comparisons were confounded. The MCC provides a balanced measure accounting for true and false positives as well as negatives from the model, even if the classes are imbalanced. The value of +1 indicates a perfect prediction, 0 indicates a prediction as good as a random and -1 indicates complete disagreement between observed and predicted class. We saw no significant improvement to the MCC when adjusting for confounders when classifying between any of the phenotypes as well as one-year follow-up (Supplementary Table 2a. 2b and 2c). Moreover, when each feature’s contribution was ranked from random forest classification, none of the tested confounders were ranked within the top 50% of features, indicating they do not contribute to classification and have no confounding effect on the measured volatilome. We used the regression coefficient (R^2^) as a regression metric and the Matthew Correlation Coefficient (MCC) as a metric to summarise the classification accuracy of our models. For participants with Parkinson’s in the UK cohort, we used random forests to determine if the measured volatilome can also determine clinical variables associated with PD (*viz*. HY stage, MOCA score, MDS_UPDRS Score, NDS, VAS, Tremor Score, PIGD Score, years since motor symptoms, years and months since clinical diagnosis and ratio between tremor and PIGD scores), medication intake or dosage of medication. We performed Pearson’s correlation analyses between medication intake and above-described clinical features and significant variables to determine if there were any associations. For participants with Parkinson’s Disease in our Austrian cohort, we used medication intake data to determine if the measured volatilome can classify PD participants by their drug intake.

## Supplementary information

Supplementary material

## Data Availability

Raw data sets generated during the current study are available from MetaboLights Repository https://www.ebi.ac.uk/metabolights/search with the study identifier MTBLS11712.
