# Supplementary material for: Classification of Parkinson’s disease and isolated REM sleep behaviour disorder: delineating progression markers from the sebum volatilome
Source: NPJ Parkinsons Dis. 2025 Jul 15;11:202. doi: 10.1038/s41531-025-01026-8 (PMC12264129; doi:10.1038/s41531-025-01026-8)
Supplement: Supplementary file 1 — Supplementary material [file 41531_2025_1026_MOESM1_ESM.docx]

**Classification of Parkinson’s Disease and idiopathic REM Sleep Behaviour Disorder: Delineating Progression Markers from the Sebum Volatilome**

Caitlin Walton-Doyle^1^, Beatrice Heim^2^, Eleanor Sinclair^1^, Sze Hway Lim^3^, Katherine A Hollywood^1^, Joy Milne^1^, Evi Holzknecht^2^, Ambra Stefani^2^, Birgit Högl^2^, Klaus Seppi^2^, Monty Silverdale^3^, Werner Poewe^2^, Perdita Barran^1*^, Drupad K Trivedi^1*^

^1^Manchester Institute of Biotechnology, Department of Chemistry, University of Manchester, Manchester, M1 7DN, UK.

^2^Department of Neurology, Innsbruck Medical University, Innsbruck, Austria.

^3^Department of Neurology, Salford Royal Foundation Trust, Manchester Academic Health Science Centre, University of Manchester, Manchester, UK, M6 8HD.

*Corresponding authors

Emails:

[drupad.trivedi@manchester.ac.uk](mailto:drupad.trivedi@manchester.ac.uk)

[perdita.barran@manchester.ac.uk](mailto:perdita.barran@manchester.ac.uk)

**Supplementary Information**

**Figure S1:** Purine was found to be upregulated in control *vs*. PD, with intermediate expression in iRBD, however was not one of the most significant markers due to low stability score (<0.8) during cross validation of models (n=100).

**
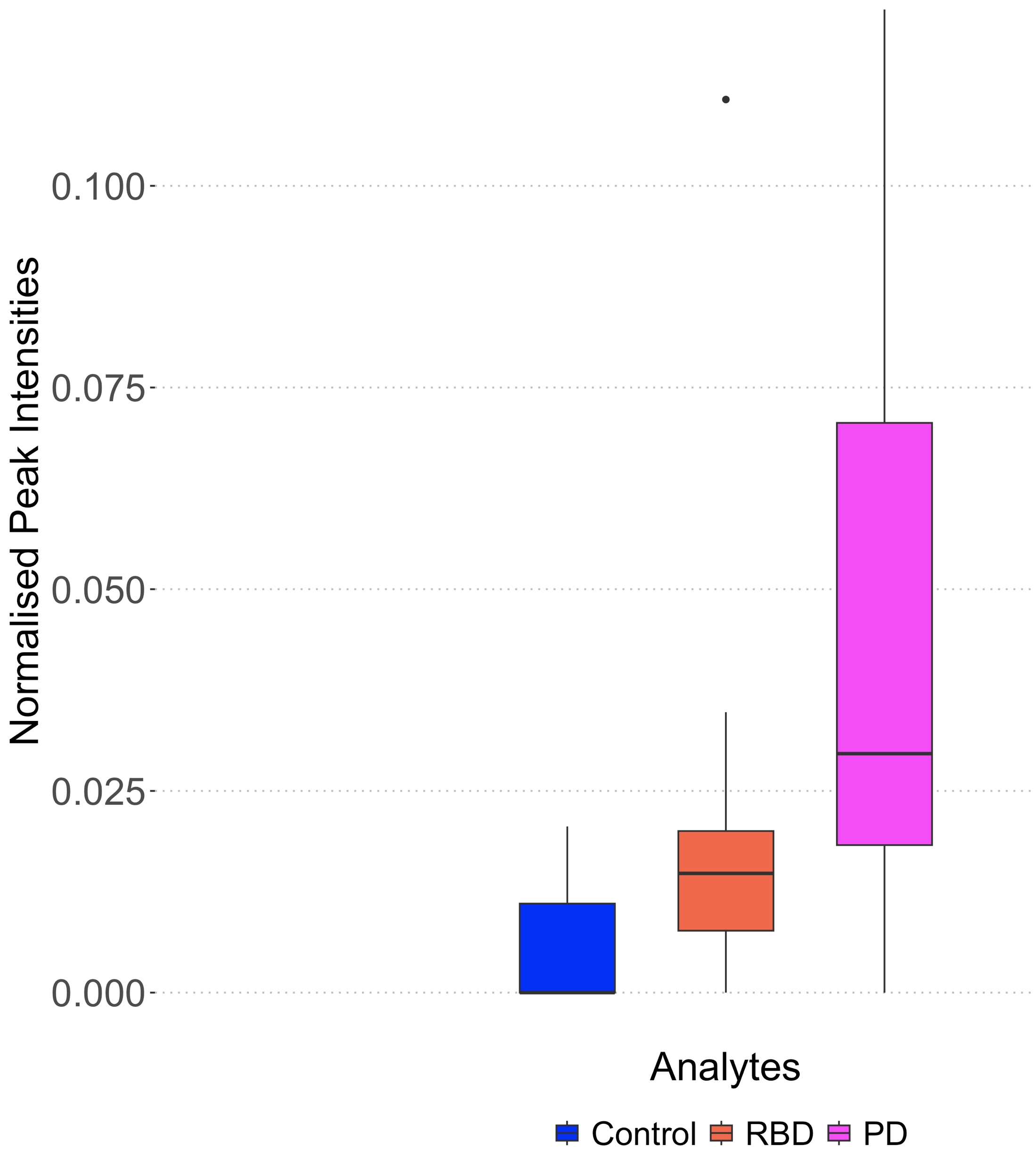
**

**Table S1: A list of features and annotations found significant in the three models**

| Feature | Putative ID | Found Significant in | | |
| --- | --- | --- | --- | --- |
|  |  | Model  PD v Control | Model  PD v RBD v Control | Model PD over 3 years |
| 5 | Oleamide | Y |  |  |
| 17 |  | Y | Y |  |
| 18 |  | Y |  |  |
| 21 |  | Y |  |  |
| 28 |  |  |  | Y |
| 29 | Alkane | Y |  |  |
| 32 |  |  |  | Y |
| 33 |  |  |  | Y |
| 39 |  |  |  | Y |
| 41 |  |  |  | Y |
| 46 |  |  |  | Y |
| 48 |  | Y |  |  |
| 51 |  | Y |  |  |
| 55 |  | Y | Y |  |
| 57 | Alkane | Y |  |  |
| 60 |  |  | Y |  |
| 61 |  |  | Y |  |
| 65 |  |  | Y |  |
| 66 |  |  | Y |  |
| 72 |  |  | Y | Y |
| 74 |  |  | Y |  |
| 75 |  | Y |  |  |
| 76 |  | Y | Y |  |
| 78 |  |  | Y |  |
| 79 |  |  | Y |  |
| 83 |  |  |  | Y |
| 89 |  | Y | Y |  |
| 90 |  |  | Y |  |
| 92 |  | Y | Y |  |
| 93 |  |  | Y |  |
| 94 |  |  | Y |  |
| 98 |  |  |  | Y |
| 102 |  | Y |  |  |
| 135 |  |  | Y |  |
| 147 |  | Y |  |  |
| 149 |  | Y |  |  |
| 150 |  | Y |  |  |
| 151 |  | Y |  |  |
| 152 |  | Y |  |  |
| 158 |  | Y | Y |  |
| 160 |  | Y |  |  |
| 176 | Alkane | Y | Y |  |
| 178 | Alkane | Y |  | Y |
| 179 |  | Y |  |  |
| 181 |  | Y |  |  |
| 186 | Alkane | Y |  |  |
| 187 | Alkane |  |  | Y |
| 190 | Alkane | Y |  |  |
| 193 | Alkane | Y |  |  |
| 195 |  | Y | Y |  |
| 197 |  | Y |  |  |
| 198 |  | Y | Y |  |
| 202 | Alkane | Y |  |  |
| 205 | Alkane | Y |  |  |
| 208 |  | Y |  |  |
| 209 |  |  | Y |  |
| 210 |  |  | Y |  |
| 215 |  | Y | Y |  |
| 223 |  |  |  | Y |
| 225 | Tropinone |  | Y | Y |
| 240 |  |  | Y |  |
| 248 | Alkane | Y | Y |  |
| 249 |  |  | Y |  |
| 251 | FAME |  | Y |  |
| 253 | Alkane |  | Y |  |
| 255 |  |  | Y |  |
| 257 | FAME |  | Y |  |
| 271 |  | Y |  |  |
| 272 | Alkane | Y | Y |  |
| 275 |  | Y | Y | Y |
| 276 |  | Y | Y |  |
| 280 |  | Y | Y |  |
| 286 |  | Y | Y |  |
| 287 |  |  | Y |  |
| 289 | Alkane | Y | Y |  |
| 295 |  | Y | Y |  |
| 302 |  |  |  | Y |
| 306 |  |  |  | Y |
| 310 |  | Y |  |  |
| 312 |  |  | Y |  |
| 315 | Alkane |  | Y |  |
| 327 | Alkane |  |  | Y |
| 328 |  |  |  | Y |
| 331 | Alkane |  | Y | Y |
| 339 |  | Y |  |  |
| 340 | Alkane |  |  | Y |
| 341 |  |  |  | Y |
| 343 | Alkane | Y | Y |  |
| 346 |  | Y |  |  |
| 347 |  | Y |  |  |
| 348 |  | Y | Y |  |
| 351 | FAME |  | Y |  |
| 353 |  | Y | Y |  |
| 354 |  |  | Y |  |
| 355 |  |  | Y |  |
| 358 |  |  | Y |  |
| 360 | Alkane | Y |  |  |
| 365 |  |  | Y |  |
| 366 |  | Y | Y |  |
| 367 | Aldehyde | Y | Y |  |
| 368 | Tropinone | Y | Y |  |
| 372 |  | Y | Y |  |
| 373 |  |  | Y |  |
| 377 | Alkane |  |  | Y |
| 378 |  |  | Y |  |
| 382 | Alkane |  | Y |  |
| 384 |  |  | Y |  |
| 387 |  |  |  | Y |
| 389 | Alkane | Y |  |  |
| 396 |  | Y |  |  |
| 397 |  | Y |  |  |
| 398 |  | Y |  |  |
| 399 | Alkane |  | Y |  |
| 400 |  |  | Y |  |
| 401 |  |  | Y |  |
| 402 |  |  |  | Y |
| 413 |  | Y | Y |  |
| 415 | Purine |  | Y |  |
| 417 |  |  | Y |  |
| 418 |  |  | Y |  |
| 423 | Alkane |  | Y |  |
| 426 |  |  |  | Y |
| 430 |  | Y |  |  |
| 432 |  | Y |  |  |
| 433 |  | Y |  |  |
| 437 |  |  |  | Y |
| 441 |  | Y |  |  |
| 444 | Alkane | Y |  |  |
| 447 |  |  | Y |  |
| 453 | FAME |  | Y | Y |
| 457 |  |  | Y |  |
| 458 | FAME | Y | Y |  |
| 459 | FAME | Y | Y |  |
| 471 |  | Y |  |  |
| 472 |  | Y |  |  |
| 473 |  | Y |  |  |
| 474 | Purine | Y |  |  |
| 477 |  | Y |  |  |
| 496 |  |  |  | Y |
| 497 |  |  |  | Y |
| 510 | Alkane |  |  | Y |
| 512 |  |  |  | Y |
| 513 |  |  |  | Y |
| 520 | FAME | Y | Y |  |
| 530 | Alkane | Y |  |  |
| 550 |  |  |  | Y |
| 551 |  | Y | Y |  |
| 552 | FAME | Y |  |  |
| 556 | FAME | Y |  |  |
| 559 |  |  | Y |  |
| 562 |  | Y |  |  |
| 564 |  | Y |  |  |
| 571 |  | Y |  |  |
| 579 | FAME | Y |  |  |
| 580 | FAME | Y | Y |  |
| 583 |  | Y |  |  |
| 584 |  | Y |  |  |
| 589 |  |  |  | Y |
| 592 |  |  |  | Y |
| 594 |  |  |  | Y |
| 603 |  |  |  | Y |
| 606 |  |  |  | Y |

**Table S2a: *MCC and correctly classified samples for unadjusted and confounder adjusted models comparing PD and Controls***

| Model | Overall MCC | Correct PD classification (%) | Correct Control classification (%) | Confounder in top 50% variables? |
| --- | --- | --- | --- | --- |
| Original | 0.992 | 100 | 99 | n/a |
| Age adjusted | 0.998 | 100 | 99.7 | No |
| BMI adjusted | 0.998 | 100 | 99.7 | No |
| Gender adjusted | 0.999 | 100 | 99.9 | No |
| Alcohol adjusted | 0.998 | 100 | 99.7 | No |
| Smoking adjusted | 0.998 | 100 | 99.7 | No |
| High cholesterol adjusted | 0.999 | 100 | 99.9 | No |
| Hypertension adjusted | 0.999 | 100 | 99.9 | No |
| Skin conditions adjusted | 0.999 | 100 | 99.9 | No |
| Bone diseases adjusted | 0.999 | 100 | 99.9 | No |
| Thyroid conditions adjusted | 0.999 | 100 | 99.9 | No |
| Heart conditions adjusted | 0.998 | 100 | 99.7 | No |

**Table S2b: *MCC and correctly classified samples for unadjusted and confounder adjusted models comparing PD, iRBD and Controls***

| Model | Overall MCC | Correct PD classification (%) | Correct Control classification (%) | Correct RBD classification (%) | Confounder in top 50% variables? |
| --- | --- | --- | --- | --- | --- |
| Original | 0.852 | 99.2 | 82.7 | 82 | n/a |
| Age adjusted | 0.868 | 98.8 | 85.3 | 84 | No |
| BMI adjusted | 0.868 | 98.7 | 85.7 | 84 | No |
| Gender adjusted | 0.889 | 99.3 | 88.3 | 85.3 | No |
| Alcohol adjusted | 0.876 | 99.2 | 86.7 | 84 | No |
| Smoking adjusted | 0.875 | 99 | 86.3 | 84.3 | No |
| High cholesterol adjusted | 0.895 | 99.5 | 89.7 | 85 | No |
| Hypertension adjusted | 0.891 | 99.5 | 89 | 84.7 | No |
| Skin conditions adjusted | 0.896 | 99.3 | 90.3 | 85 | No |
| Bone diseases adjusted | 0.888 | 99.3 | 89 | 84.3 | No |
| Thyroid conditions adjusted | 0.891 | 99.3 | 89 | 85 | No |
| Heart conditions adjusted | 0.898 | 99.3 | 90.7 | 85 | No |

**Table S2c: *MCC and correctly classified samples for unadjusted and confounder adjusted models comparing PD participant at recruitment and at one year follow up.***

| Model | Overall MCC | Correct PD Year 1 classification (%) | Correct PD Year 2 classification (%) | Confounder in top 50% variables? |
| --- | --- | --- | --- | --- |
| Original | 0.833 | 92.5 | 90.8 | n/a |
| Levodopa LED adjusted | 0.833 | 92.5 | 90.8 | No |
| LLED (mg) adjusted | 0.833 | 92.5 | 90.8 | No |
| MDS_UPDRS Score adjusted | 0.833 | 92.5 | 90.8 | No |
| MOCA Score adjusted | 0.833 | 92.5 | 90.8 | No |
| HY stage adjusted | 0.838 | 92.5 | 91.2 | No |

**Table S3: *For classification and regression models when confounder used as outcome, Matthew Correlation Coefficient (MCC) and R2, used as performance indicators.***

|  | PD vs Control | PD v iRBD v Control |
| --- | --- | --- |
| Gender MCC | 0.345 | -0.063 |
| High cholesterol MCC | 0.067 | -0.009 |
| Hypertension MCC | 0.015 | 0.002 |
| Skin condition MCC | -0.011 | n/a* |
| Bone disease MCC | -0.028 | -0.051 |
| Thyroid condition MCC | 0 | -0.039 |
| Heart condition MCC | 0 | -0.021 |
| Age R2 | -0.091 | -0.222 |
| BMI R2 | -0.107 | -0.214 |
| Alcohol R2 | -0.244 | -0.193 |
| Smoking R2 | -0.154 | -0.125 |

**indicates not sufficient samples per group to perform analysis.*

**Table S4: Prediction of clinical characteristic and drug dosage/intake within PD cohort (from Manchestger cohort the data taken is from visit one only) using measured volatilome. MCC and R^2^ used for model performance measurement.**

| Clinical characteristic or medication | Longitudinal PD comparison | PD only UK | PD only UK and Austria |
| --- | --- | --- | --- |
| HY Stage MCC | 0.137 | -0.04 | n/a |
| MOCA Score R^2^ | -0.174 | -0.099 | n/a |
| MDS_UPDRS Score R^2^ | -0.307 | -0.261 | n/a |
| LLED (mg) R^2^ | -0.455 | -0.304 | n/a |
| Levodopa dosage R^2^ | -0.493 | -0.286 | n/a |
| Amantadine MCC | n/a | 0 | -0.019 |
| Pramipexole MCC | n/a | -0.045 | -0.009 |
| Ropinirole MCC | n/a | -0.017 | -0.037 |
| Rotigotone MCC | n/a | 0 | 0 |
| Rasagiline MCC | n/a | 0.284 | 0.182 |
| Entacapone MCC | n/a | n/a* | -0.023 |
| L-dopa MCC | n/a | -0.113 | 0.328 |
| Comorbidity MCC | n/a | 0.143 | n/a |
| NDS R^2^ | n/a | -0.093 | n/a |
| VAS R^2^ | n/a | -0.228 | n/a |
| mins since last medication R^2^ | n/a | -0.541 | n/a |
| Tremor Score R^2^ | n/a | -0.16 | n/a |
| PIGD Score R^2^ | n/a | -0.314 | n/a |
| Tremor/PIGD ratio R^2^ | n/a | -0.258 | n/a |
| Years of PD symptoms R^2^ | n/a | -0.442 | n/a |
| Years since diagnosis R^2^ | n/a | -0.398 | n/a |
| Months since diagnosis R^2^ | n/a | -0.398 | n/a |
| Tremor or PIGD or Intermediate MCC | n/a | -0.211 | n/a |

*n/a indicates no data available for the cohort and * indicates not sufficient data for samples in each class for analysis.*
